# Hydrogen Peroxide Pretreatment Mitigates Cadmium-Induced Oxidative Stress in *Brassica napus* L.: An Intrinsic Study on Antioxidant Defense and Glyoxalase Systems

**DOI:** 10.3389/fpls.2017.00115

**Published:** 2017-02-10

**Authors:** Mirza Hasanuzzaman, Kamrun Nahar, Sarvajeet S. Gill, Hesham F. Alharby, Bam H. N. Razafindrabe, Masayuki Fujita

**Affiliations:** ^1^Department of Agronomy, Faculty of Agriculture, Sher-e-Bangla Agricultural UniversitySher-e-Bangla Nagar, Bangladesh; ^2^Department of Agricultural Botany, Faculty of Agriculture, Sher-e-Bangla Agricultural UniversitySher-e-Bangla Nagar, Bangladesh; ^3^Laboratory of Plant Stress Responses, Department of Applied Biological Science, Faculty of Agriculture, Kagawa UniversityMiki-cho, Japan; ^4^Stress Physiology and Molecular Biology Laboratory, Centre for Biotechnology, Maharshi Dayanand UniversityRohtak, India; ^5^Department of Biological Sciences, Faculty of Science, King Abdulaziz UniversityJeddah, Saudi Arabia; ^6^Department of Subtropical Agro-Environmental Sciences, Faculty of Agriculture, University of the RyukyusNishihara, Japan

**Keywords:** abiotic stress, antioxidant defense, cross tolerance, metal toxicity, methylglyoxal, oxidative stress, signaling molecule

## Abstract

Cadmium (Cd) is considered as one of the most toxic metals for plant growth and development. In the present study, we investigated the role of externally applied hydrogen peroxide (H_2_O_2_) in regulating the antioxidant defense and glyoxalase systems in conferring Cd-induced oxidative stress tolerance in rapeseed (*Brassica napus* L.). Seedlings were pretreated with 50 μM H_2_O_2_ for 24 h. These pretreated seedlings as well as non-pretreated seedlings were grown for another 48 h at two concentrations of CdCl^2^ (0.5 and 1.0 mM). Both the levels of Cd increased MDA and H_2_O_2_ levels and lipoxygenase activity while ascorbate (AsA) declined significantly. However, reduced glutathione (GSH) content showed an increase at 0.5 mM CdCl^2^, but glutathione disulfide (GSSG) increased at any level of Cd with a decrease in GSH/GSSG ratio. The activities of ascorbate peroxidase (APX) and glutathione *S*-transferase (GST) upregulated due to Cd treatment in dose-dependent manners, while glutathione reductase (GR) and glutathione peroxidase (GPX) increased only at 0.5 mM CdCl^2^ and decreased at higher dose. The activity of monodehydroascorbate reductase (MDHAR), dehydroascorbate reductase (DHAR), catalase (CAT), glyoxalase I (Gly I), and glyoxalase II (Gly II) decreased under Cd stress. On the other hand, H_2_O_2_ pretreated seedlings, when exposed to Cd, AsA and GSH contents and GSH/GSSG ratio increased noticeably. H_2_O_2_ pretreatment increased the activities of APX, MDHAR, DHAR, GR, GST, GPX, and CAT of Cd affected seedlings. Thus enhancement of both the non-enzymatic and enzymatic antioxidants helped to decrease the oxidative damage as indicated by decreased levels of H_2_O_2_ and MDA. The seedlings which were pretreated with H_2_O_2_ also showed enhanced glyoxalase system. The activities of Gly I, and Gly II and the content of GSH increased significantly due to H_2_O_2_ pretreatment in Cd affected seedlings, compared to the Cd-stressed plants without H_2_O_2_ pretreatment which were vital for methylglyoxal detoxification. So, the major roles of H_2_O_2_ were improvement of antioxidant defense system and glyoxalase system which protected plants from the damage effects of ROS and MG. The mechanism of H_2_O_2_ to induce antioxidant defense and glyoxalase system and improving physiology under stress condition is not known clearly which should be elucidated. The signaling roles of H_2_O_2_ and its interaction with other signaling molecules, phytohormones or other biomolecules and their roles in stress protection should be explored.

## Introduction

Metal toxicity has been increasing considerably due to increase of toxic metal release as a result of urbanization and industrialization (Hasanuzzaman and Fujita, 2012). Cadmium (Cd) is considered as most toxic considering injurious effects on plant developmental processes and metabolism (Nouairi et al., 2009). Cd has nature to enter through roots readily and easily due to its high solubility in water. Cd content higher than 5–10 μg Cd g^-1^ leaf dry weight is considered toxic for plants, in general (White and Brown, 2010). Cd stress reduces growth and metabolism affecting plants’ basic physiological processes including water and nutrient translocation and assimilation, transpiration and photosynthesis (Hasanuzzaman and Fujita, 2012; Khan et al., 2015). At cellular level Cd provokes generation of ROS [may include superoxide anion ($O_{2}^{•–}$), hydroxyl radical (^∙^OH), alkoxyl (RO^∙^), peroxyl (ROO^∙^), hydrogen peroxide (H_2_O_2_), singlet oxygen (^1^O_2_), and so forth] which results in oxidative damages to lipids, proteins and fatty acid which disrupt biomembrane, ultrastructural cellular components, DNA, and causes programmed cell death (PCD; Gill and Tuteja, 2010; Nahar et al., 2015, 2016a). Plants’ antioxidant system scavenges ROS and keeps a state of balance under non-stress condition. Antioxidant machinery posses non-enzymatic antioxidants [AsA, GSH, flavonoids, phenolic compounds, carotenoids, alkaloids, Pro, non-protein amino acids, and α-tocopherols] and a bunch of antioxidant enzymes [CAT, APX, MDHAR, DHAR, GR, GPX, guaiacol peroxidase, and GST] which works in coordinated manner to scavenge ROS and to minimize oxidative stress (Apel and Hirt, 2004; Hasanuzzaman et al., 2012b). MG generation is an impulsive outcome of the glycolysis. Due to environmental stresses MG is overproduced many times higher than the normal growth condition to create toxic effects (Kaur et al., 2015). In glyoxalase system, utilizing GSH MG is transformed in to SLG by the activity of Gly I, while Gly II transforms SLG to D-lactic acid which is a MG detoxification process. At the end, GSH is regenerated. Tolerance against ROS and MG confers and improves abiotic stress adaptation and tolerance in different plants (Yadav et al., 2008; Hasanuzzaman et al., 2012a; Kaur et al., 2015).

Among the ROS, H_2_O_2_ has stability, being a versatile molecule shows signaling function (Quan et al., 2008; Saxena et al., 2016). It takes part of oxidative metabolism. It has been proved to involve in signaling cascades and metabolism which are vital for plants growth/developmental processes. Seed germination, initiation of root hair, strengthening of cell wall, cell wall loosening, xylem differentiation and stomatal movement were reported to link with H_2_O_2_ mediated signaling cascade (Dempsey and Klessig, 1995; Wojtyla et al., 2016). Interacting with other hormones and signaling molecules [abscisic acid (ABA) and ethylene], H_2_O_2_ regulates plant metabolism (Jubany-Mari et al., 2009; Chen et al., 2012). Recently it has been reported that nitric oxide (NO) and H_2_O_2_ regulate the salicylic acid (SA)- induced salvianolic acid B production (Guo et al., 2014). Thus, as a signaling molecule H_2_O_2_ regulates different metabolic pathways to develop stress tolerances (Mittler et al., 2004; Reczek and Chandel, 2015). H_2_O_2_-induced signal stimulates the expression and activation of stress tolerant genes (Prasad et al., 1994) which mediate stress acclimation and adaptation (Uchida et al., 2002). In different research findings, H_2_O_2_ mediated chilling (Prasad et al., 1994), salinity (Xu et al., 2008; Li et al., 2011), heat (Gao et al., 2010), osmotic stress (Liu et al., 2010), Cd (Hu et al., 2009), low light (Zhang et al., 2011), and multiple stress (Gong et al., 2001) tolerances were reported. Based on the results of previous studies we hypothesize that application of exogenous H_2_O_2_ might have a signaling function, influence antioxidant activities which can improve Cd stress tolerance. Very few research works demonstrated the beneficial roles of H_2_O_2_ on Cd or heavy metal stress (Chao et al., 2009; Hu et al., 2009). In the previous study, only few components of antioxidant defense system have been examined to show the effect of H_2_O_2_ under Cd stress (Chao et al., 2009; Hu et al., 2009). Moreover, effects of H_2_O_2_ on MG detoxification system under Cd stress were not reported. Many aspects of H_2_O_2_-induced Cd stress tolerance are yet to be elucidated. The present study provides a new insight into H_2_O_2_-induced coordinated effects on antioxidant defense and glyoxalase system to enhance the resistance to Cd toxicity in rapeseed seedlings. In this study, we will present several components of antioxidant defense and MG detoxification systems which were not mentioned in previous research findings.

## Materials and Methods

### Plant Material, Growth Condition, and Treatments

Healthy and uniform sized rapeseed (*Brassica napus* cv. BINA sharisha 3) seeds were dipped into 70% ethanol for 5 min, then washed with double distilled water (ddH_2_O). Seeds had been sown in Petri plates (9 cm) containing six layers of filter paper where filter papers were provided with 10 ml of ddH_2_O. The Petridishes containing seeds were kept in a dark germination chamber under controlled conditions, 72 h. Germinated seedlings were removed from the germinator and placed into growth chamber under control environment (providing with light 100 μmol photon m^-2^ s^-1^, temp 25 ± 2°C, RH 65–70%). Seedlings were supplied with 10,000-fold diluted Hyponex solution (Hyponex, Japan) as nutrient at regular interval. Eleven-day-old seedlings were pretreated with 50 μM H_2_O_2_ in their root for 24 h. Both H_2_O_2_-pretreated and non-pretreated seedlings were then exposed to Cd stress (0.5 and 1.0 mM CdCl_2_) for 48 h. Several trial experiments were conducted before selecting the present doses of treatments. Different doses of Cd were applied in combination with different doses of H_2_O_2_ and the present combination (0.5 and 1.0 mM CdCl_2_ with 50 μM H_2_O_2_; 48 h) showed the better result. We hypothesized that using two concentrations of Cd the trend how the H_2_O_2_ is affecting the Cd-stressed rapeseed seedlings could be understood better. The same experiment was repeated three times under the same treatment condition. There were 45 seedlings in each Petri dish. In total 6 × 3 = 18 dishes were used.

### Measurement of Lipid Peroxidation

Lipid peroxidation had been determined by estimating MDA (a product of lipid peroxidation) using TBA (Heath and Packer, 1968; Hasanuzzaman et al., 2011).

### Measurement of Hydrogen Peroxide Content

Hydrogen peroxide (H_2_O_2_) had been determined extracting leaves in potassium phosphate (K-P) buffer (pH 6.5; centrifuging at 11,500×*g*), then adding it to a mixture of TiCl_4_ in 20% H_2_SO_4_ (v/v). The supernatant was read spectrophotometrically at 410 nm (Yu et al., 2003).

### Histochemical Detection of Hydrogen Peroxide and Superoxide

The H_2_O_2_ and $O_{2}^{•–}$ were determined histochemically (Chen et al., 2010) in the leaves of rapeseed plants by staining leaves with 1% 3,3-diaminobenzidine (DAB; to get brown spots due to the reaction of DAB with H_2_O_2_) and 0.1% nitroblue tetrazolium chloride (NBT; to get deep blue spots appeared due to the reaction of NBT with $O_{2}^{•–}$) solution, respectively. Then, leaves were blanched in boiling ethanol to visualize the spots.

### Extraction and Measurement of Ascorbate and Glutathione

The leaves of rapeseed plant (0.5 g) had been homogenized in 5% meta-phosphoric acid containing 1 mM EDTA (centrifuged at 11,500 × *g*; 15 min at 4°C). Supernatant was collected for the assay of AsA and GSH pool. To determine total ascorbate, the oxidized fraction was reduced by adding 0.1 M dithiothreitol for 1 h at room temperature and then read at 265 nm using 1.0 unit AO. Oxidized ascorbate (DHA) content had been assayed by subtracting reduced AsA from total AsA (Hasanuzzaman et al., 2011; Nahar et al., 2016b). The glutathione pool had been determined according to previously described methods (Yu et al., 2003; Hasanuzzaman et al., 2011). Standard curves with known concentrations of GSH and GSSG had been used to calculate the unknown GSH and GSSG pool of plant sample. The content of reduced GSH had been calculated by subtracting GSSG from total GSH.

### Protein Determination

Following the method of Bradford (1976) the protein content had been measured where we used BSA as a protein standard.

### Enzyme Extraction and Assays

Leaves had been homogenized with 50 mM K-P buffer (pH 7.0) containing 100 mM KCl, 1 mM AsA, 5 mM β-mercaptoethanol, and 10% (w/v) glycerol in pre-chilled mortars. Homogenates were centrifuged at 11,500 × *g*. The supernatants were collected and used for the assay of enzyme activity.

Ascorbate peroxidase (EC: 1.11.1.11) activity: The reaction buffer solution contained 50 mM K-P buffer (pH 7.0), 0.5 mM AsA, 0.1 mM H_2_O_2_, 0.1 mM EDTA, and enzyme extract (final volume 700 μL). The reaction had been initiated adding H_2_O_2_. Absorbance had been monitored at 290 nm for 1 min and activity has been calculated using an extinction coefficient of 2.8 mM^-1^cm^-1^ (Nakano and Asada, 1981).

Monodehydroascorbate reductase (EC: 1.6.5.4) activity: The reaction mixture contained 50 mM Tris-HCl buffer (pH 7.5), 0.2 mM NADPH, 2.5 mM AsA, 0.5 unit of AO, and enzyme solution (final volume 700 μL). The reaction had been started by adding AO. Absorbance was taken at 340 nm; activity had been calculated from the change in absorbance for 1 min using an extinction coefficient of 6.2 mM^-1^cm^-1^ (Hossain et al., 1984).

Dehydroascorbate reductase (EC: 1.8.5.1) activity: The reaction buffer contained 50 mM K-P buffer (pH 7.0), 2.5 mM GSH, and 0.1 mM DHA. Activity had been calculated from the change in absorbance at 265 nm for 1 min using an extinction coefficient of 14 mM^-1^cm^-1^ (Nakano and Asada, 1981).

Glutathione reductase (EC: 1.6.4.2) activity: The reaction mixture contained 0.1 M K-P buffer (pH 7.0), 1 mM EDTA, 1 mM GSSG, 0.2 mM NADPH, and enzyme solution (final volume 1 mL). The reaction had been started with GSSG; the decrease in absorbance at 340 nm was monitored for 1 min and activity had been calculated using an extinction coefficient of 6.2 mM^-1^cm^-1^ (Hasanuzzaman et al., 2011).

Glutathione *S*-transferase (EC: 2.5.1.18) activity: The reaction mixture had 100 mM Tris-HCl buffer (pH 6.5), 1.5 mM GSH, 1 mM CDNB, and enzyme solution (final volume 700 μL). The reaction had been started by CDNB; the raise of absorbance was monitored at 340 nm for 1 min. Activity had been calculated using an extinction coefficient of 9.6 mM^-1^cm^-1^ (Hossain et al., 2006).

Glutathione peroxidase (EC: 1.11.1.9) activity: The reaction mixture contained of 100 mM K-P buffer (pH 7.0), 1 mM EDTA, 1 mM NaN_3_, 0.12 mM NADPH, 2 mM GSH, 1 unit GR, 0.6 mM H_2_O_2_ (as a substrate), and 20 μL of sample solution. The oxidation of NADPH had been observed at 340 nm for 1 min and the activity was calculated using an extinction coefficient of 6.62 mM^-1^cm^-1^ (Hasanuzzaman et al., 2011).

Catalase (EC: 1.11.1.6) activity: Decrease of absorbance (by decomposition of H_2_O_2_) at 240 nm had been noticed for 1 min. The reaction had been started with enzyme extract; activity has been calculated using an extinction coefficient of 39.4 M^-1^cm^-1^ (Hasanuzzaman et al., 2011).

Glyoxalase I (EC: 4.4.1.5): The assay mixture contained 100 mM K-P buffer (pH 7.0), 15 mM magnesium sulfate, 1.7 mM GSH, and 3.5 mM MG (final volume 700 μL). Adding MG the reaction had been started; the increase in absorbance was recorded at 240 nm for 1 min. Activity had been calculated using an extinction coefficient of 3.37 mM^-1^cm^-1^ (Hasanuzzaman et al., 2011).

Glyoxalase II (EC: 3.1.2.6): Formation of GSH was monitored for 1 min at 412 nm. The reaction mixture contained 100 mM Tris-HCl buffer (pH 7.2), 0.2 mM DTNB, and 1 mM SLG (final volume of 1 mL). Reaction had been initiated by adding SLG; activity had been calculated using an extinction coefficient of 13.6 mM^-1^cm^-1^ (Principato et al., 1987).

Lipoxygenase (EC 1.13.11.12): LOX activity was estimated monitoring the increase of absorbance at 234 nm using linoleic acid as a substrate. Activity had been calculated using an extinction coefficient of 25 mM^-1^cm^-1^ and expressed as units (1 nmol of substrate oxidized per min) mg^-1^ protein (Doderer et al., 1992).

### Statistical Analysis

All data were subjected to analysis of variance (ANOVA). Mean differences had been compared by Tukey’s HSD test using XLSTAT v. 2016.04.32525 software (Addinsoft, 2016). Differences at *P* ≤ 0.05 were considered significant.

## Results

### Production of ROS and Oxidative Stress

**FIGURE 1:**
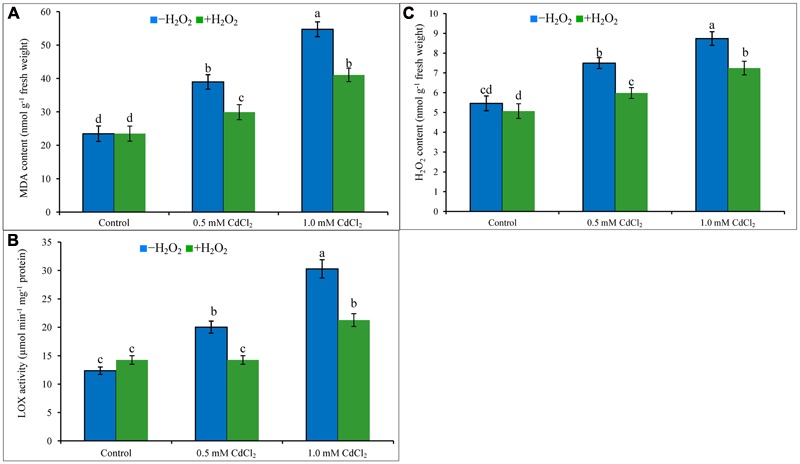
**Malondialdehyde content (A)**, H_2_O_2_ content **(B)**, and LOX activity **(C)** in rapeseed leaves induced by exogenous H_2_O_2_ under Cd stress. Mean (±SD) was calculated from three replicates for each treatment. Bars with different letters are significantly different at *P* < 0.05 applying Tukey’s HSD test.

Cadmium stress imposition in the growing media caused oxidative damage in the seedlings. Membrane lipid peroxidation (increasing MDA levels) has been noticed in Cd-affected rapeseed seedlings (**Figure 1A**). Content of H_2_O_2_ increased by 37 and 60%, and activity of LOX increased by 62 and 145% under 0.5 and 1 mM CdCl_2_ stresses (**Figures 1B,C**), respectively, as compared with control plants. All these were responsible for peroxidation of membrane lipid. Exogenous H_2_O_2_ application reduced H_2_O_2_ content and LOX activity which are corroborating with the reduction of MDA contents by 23 and 25% in mild and severe Cd stresses when compared to stress treatments only (**Figures 1A–C**).

### Histochemical Detection of ROS in Rapeseed Leaves

**FIGURE 2:**
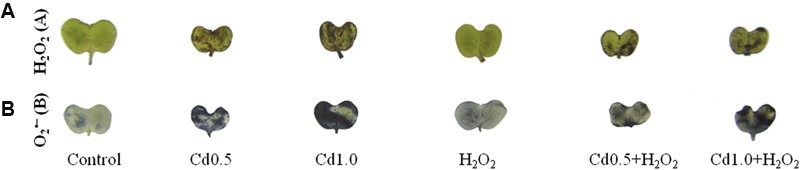
**3,3-Diaminobenzidine staining (A)** of H_2_O_2_ and NBT staining **(B)** of superoxide $O_{2}^{•–}$ in rapeseed leaves induced by exogenous H_2_O_2_ under Cd stress.

Leaves were dipped into DAB and NBT solution to visualize the generation and spots of H_2_O_2_ and $O_{2}^{•–}$, respectively. The leaves under Cd stress showed a high frequency of dark brown patches of H_2_O_2_ and deep blue spots of $O_{2}^{•–}$ anions (**Figures 2A,B**). The spots were darker and larger in severe Cd stress, compared to the mild Cd stress. However, these spots of H_2_O_2_ and $O_{2}^{•–}$ were somewhat reduced, compared to Cd stress alone when exogenous H_2_O_2_ was added with Cd stresses which are indicators for oxidative stress reduction (**Figures 2A,B**).

### ASA-GSH Cycle

**FIGURE 3:**
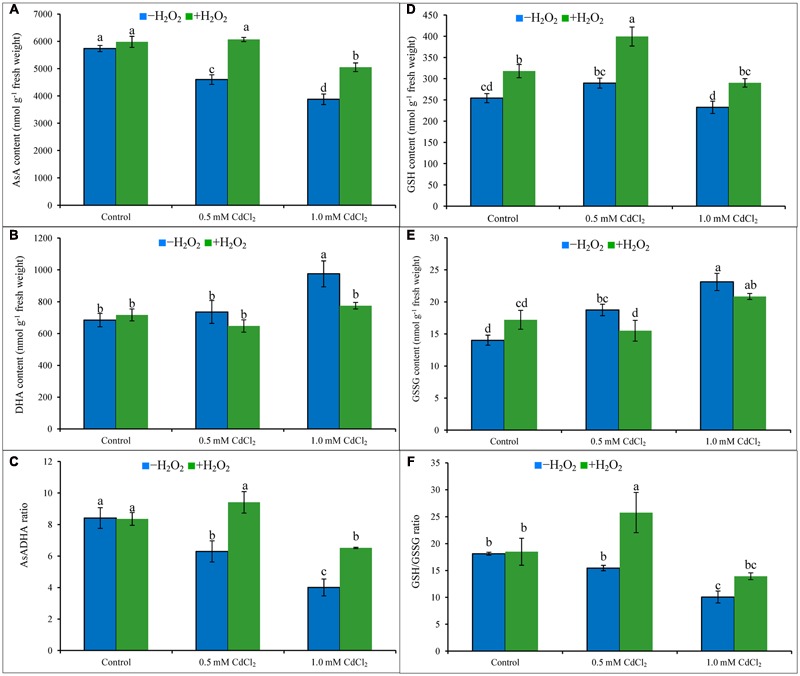
**Ascorbate (AsA) content (A)**, DHA content **(B)**, AsA/DHA ratio **(C)**, GSH content **(D)**, GSSG content **(E)**, and GSH/GSSG ratio **(F)** in rapeseed leaves induced by exogenous H_2_O_2_ under Cd stress. Mean (±SD) was calculated from three replicates for each treatment. Bars with different letters are significantly different at *P* < 0.05 applying Tukey’s HSD test.

Ascorbate content decreased by 20 and 32%; in contrast, DHA content increased by 7 and 43% which resulted in 25 and 52% decrease of AsA/DHA ratio under mild and severe Cd stresses when compared to Cd untreated control. Increase of GSH pool and also with the high increase of GSSG resulted in decreased ratio of GSH/GSSG by 15 and 44%, respectively, under 0.5 and 1 mM CdCl_2_ stress, respectively, compared to control. Exogenous H_2_O_2_ addition inverted the AsA-GSH pool by increasing AsA content by 32 and 30% (**Figure 3A**), increasing GSH content by 38 and 25% (**Figure 3D**), decreasing DHA content by 12 and 21% (**Figure 3B**), and decreasing GSSG content by 17 and 8% (**Figure 3E**), under mild and severe Cd stresses, respectively. Alteration of AsA and GSH contents by H_2_O_2_ pretreatment were vital in improving AsA/DHA (**Figure 3C**) and GSH/GSSG (**Figure 3F**) ratios, compared to Cd stress alone.

**FIGURE 4:**
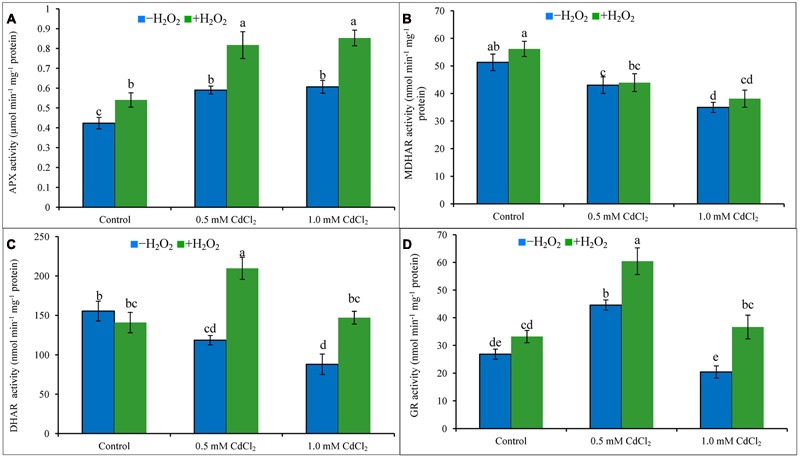
**Activities of AsA-GSH cycle enzymes, APX (A)**, MDHAR **(B)**, DHAR **(C)**, and GR **(D)** in rapeseed leaves induced by exogenous H_2_O_2_ under Cd stress. Mean (±SD) was calculated from three replicates for each treatment. Bars with different letters are significantly different at *P* < 0.05 applying Tukey’s HSD test.

The enzymes [APX, MDHAR, DHAR, and GR] (**Figures 4A–D**) of AsA-GSH cycle responded differentially in Cd-exposed seedlings. APX activity increased, MDHAR and DHAR activities reduced with the increase of Cd dose, compared to control, whereas, GR activity increased under mild Cd stress but reduced under severe Cd stress in comparison to their respective control (**Figures 4A–D**). External application of H_2_O_2_ under Cd stress increased activities of APX (40 and 39%), DHAR (77 and 67%), and GR (36 and 79%), respectively, under mild and severe Cd stresses, respectively, in contrast to Cd stress alone (**Figures 4A–D**).

### CAT, GPX, and GST Activities

**FIGURE 5:**
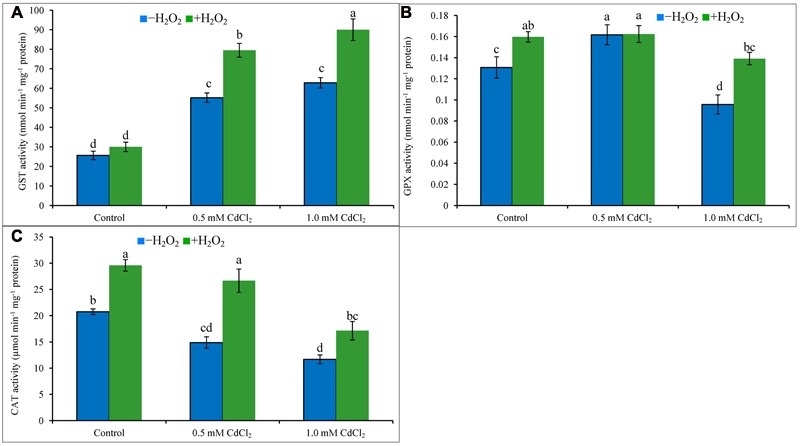
**Activities of GPX (A)**, GST **(B)**, and CAT **(C)** in rapeseed leaves induced by exogenous H_2_O_2_ under Cd stress. Mean (±SD) was calculated from three replicates for each treatment. Bars with different letters are significantly different at *P* < 0.05 applying Tukey’s HSD test.

Both levels of Cd stress-affected seedlings showed higher GST activity whereas GPX activity increased only under mild Cd stress level, but CAT activity decreased at both levels of Cd stresses when compared to control. Activity of GST upregulated by 115 and 145%, the activities of CAT reduced by 28 and 44% under mild and severe Cd stress, respectively; activity of GPX amplified by 23% under mild Cd stress but it decreased by 23% under severe Cd stress, compared to control (**Figures 5A–C**). Supplementation of H_2_O_2_ with Cd improved GST activities by 44 and 43%, and CAT activities by 79 and 47%, under mild and severe Cd stresses whereas augmented GPX activity by 40% under severe stress (**Figures 5A–C**), respectively, compared to Cd stress alone.

### Glyoxalase System

**FIGURE 6:**
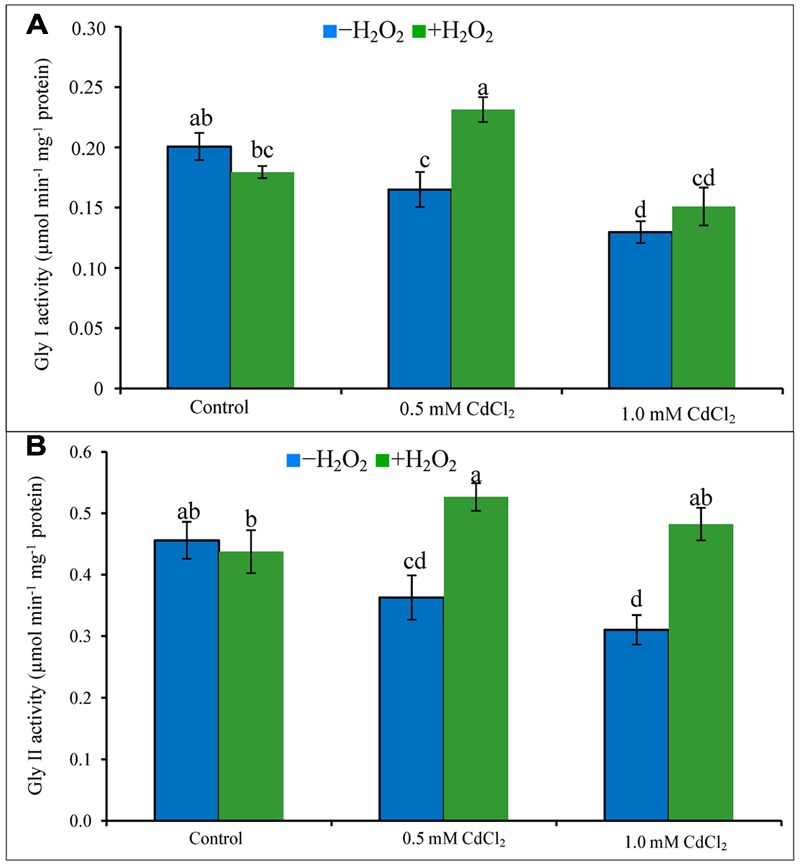
**Activities of Gly I (A)** and Gly II **(B)** in rapeseed leaves induced by exogenous H_2_O_2_ under Cd stress. Mean (±SD) was calculated from three replicates for each treatment. Bars with different letters are significantly different at *P* < 0.05 applying Tukey’s HSD test.

Activities of Gly I and Gly II decreased due to exposure of Cd (**Figures 6A,B**). Their activities increased in both doses of Cd stress treatments supplemented with H_2_O_2_ except for Gly I activity at severe stress. The increase of Gly I activity under mild Cd stress was 35% after H_2_O_2_ supplementation, compared to Cd stress alone. Gly II activity increased by 47 and 55% in H_2_O_2_ added mild and severe Cd stresses, compared to Cd stress alone (**Figures 6A,B**).

## Discussion

Showing toxicity at higher concentration and acting as signaling molecule initiate, H_2_O_2_ plays a dual role and is considered as rival and comrade of stress tolerance development in plants. Due to dual roles of H_2_O_2_ and due to various unidentified roles of H_2_O_2_, recent research with H_2_O_2_ concentrate on diversified plausible mechanisms through which H_2_O_2_ is related to plant stress tolerance development. Present study has been executed to reveal the pivotal roles of H_2_O_2_ in relation to Cd stress tolerance in rapeseed.

The mechanism of Cd-induced oxidative stress is different from other stresses; Cd^2+^ cannot produce ROS directly as it through Fenton reaction or Haber Weiss reaction. Showing affinity to thiol Cd run downs GSH (Lopez et al., 2006). Cd enhances ROS production by weakening antioxidant defense mechanism (Srivastava et al., 2004; Gill and Tuteja, 2010), distressing photosystem II activity (Sigfridsson et al., 2004), disturbing functioning of vital enzymes (Dong et al., 2006). Cd displaces iron (Fe) from proteins and increases free Fe that is responsible for ROS generation. Cd also increases ROS production distorting mitochondrial function (Dorta et al., 2003). Spots of H_2_O_2_ and $O_{2}^{•–}$ in leaves of rapeseed and the contents of H_2_O_2_ and MDA have been increased considerably in Cd affected rapeseed plants clearly indicating oxidative damage corroborating the results of previous studies (Dong et al., 2006; Hu et al., 2009; Hasanuzzaman et al., 2012a). H_2_O_2_ pretreatment reduced oxidative damage by decreasing the spots of $O_{2}^{•–}$ and H_2_O_2_ and reducing the amount of H_2_O_2_ and MDA contents against Cd toxicity (Hu et al., 2009), reducing contents of MDA and $O_{2}^{•–}$ in salinity affected wheat plants (Li et al., 2011), decreasing $O_{2}^{•–}$, H_2_O_2_ and MDA in chill affected cucumber seedlings (Zhang et al., 2011). The results of these previous reports indicate the decisive functions of H_2_O_2_ in reducing oxidative stress. At low concentration, H_2_O_2_ can as signaling molecule which modulates various genes related to stress defense mechanism. H_2_O_2_ implicated NO-mediated ABA-induced activation of mitogen-activated protein (MAP) kinase cascade which modulated antioxidant defense mechanism maize leaves. H_2_O_2_ can modulate NO and NO itself is an ROS scavenger (Zhang et al., 2007). In present study, the advantageous roles of H_2_O_2_ have been presented in later section where application of very low concentration exogenous H_2_O_2_ pretreatment induced and enhanced the antioxidant defense system components which in turn helped in decreasing the endogenous contents of ROS including H_2_O_2_ and $O_{2}^{•–}$ and in decreasing the oxidative damage which is parallel with the results of the previous findings (Zhang et al., 2007, 2011). Both AsA and GSH presenting in chloroplast, cytoplasm, apoplast, mitochondria, peroxisome effectively scavenge H_2_O_2_. CAT, APX, GPX, and GST directly catalyze the reactions of H_2_O_2_ scavenging. Exogenous low dose of H_2_O_2_ in the present study enhanced the activities of these enzymes and increased the contents of AsA and GSH of Cd affected rapeseed seedlings which are directly related to H_2_O_2_ scavenging process and that is why H_2_O_2_ pretreatment decreased the endogenous H_2_O_2_ levels and subsequent oxidative damage of Cd affected seedlings (Mittler, 2002; Blokhina et al., 2003; Ashraf, 2009; Gill and Tuteja, 2010).

Ascorbate is water-soluble non-enzymatic antioxidant in cell decreasing oxidative stress scavenging $O_{2}^{•–}$ and OH^∙^ (Gill and Tuteja, 2010). In rapeseed seedlings of present study, AsA level reduced and DHA level increased (because AsA is oxidized to DHA after scavenging ROS) due to reduced MDHAR and DHAR activities (which are AsA recycling enzymes) which decreased the AsA/DHA ratio and increased oxidative stress (Chao et al., 2010). APX activity upregulated due to Cd exposure, which is correlated to the reduced AsA content. H_2_O_2_ pretreatment followed by Cd exposure upregulated APX, MDHAR and DHAR activities in the seedlings which restored AsA and decreased oxidative stress which is supported by previous findings (Hu et al., 2009; Li et al., 2011; Zhang et al., 2011). In rapeseed seedlings, increased levels of GSH and GSSG but decreased GSH/GSSG ratio have been noticed in exposure to Cd which are supported by previous studies (Molina et al., 2008; Hu et al., 2009; Hasanuzzaman et al., 2012a). GSH is the thiol group of non-enzymatic antioxidant showing an imperative role in the stress signal, adaptation and defense mechanism of plants (Noctor et al., 2012). GSSG is recycled to GSH involving the GR activity (Gill and Tuteja, 2010). Increased GSH content after H_2_O_2_ application was found beneficial under Cd stress (Chao et al., 2009). The application of H_2_O_2_ upregulated AsA and GSH levels and improved CAT, POD, SOD, GPX, GR, MDHAR, and DHAR metabolism as reported in Al affected wheat seedlings (Xu et al., 2010). Seedlings pretreated with H_2_O_2_ increased GR activity (which recycles GSSG to GSH) which resulted in decreased GSSG level and increased GSH content which increased GSH/GSSG ratio in Cd affected rapeseed seedlings which is supported by previous findings (Hu et al., 2009; Li et al., 2011; Zhang et al., 2011).

The multifunctional isoenzymes GSTs are vital antioxidant enzymes, involved xenobiotic and toxic compound detoxification process (Polidoros and Scandalios, 1999). In the present investigation, GST activity and GSH amplified due to Cd exposure which were also observed in other studies (Hu et al., 2009; Hasanuzzaman et al., 2012a). Nonetheless, a further increase of GST activity and GSH content was noticed in H_2_O_2_ pretreated rapeseed seedlings under Cd stress which reduced adverse effects of Cd on physiology and growth which is similar to the findings of Hu et al. (2009) in rice. Increased Cd sequestration by H_2_O_2_ pretreatment in rice roots is an indication of crucial roles of H_2_O_2_ to further decline of Cd translocation to shoot (Hu et al., 2009). Cd stress reduced CAT and GPX activities in rapeseed seedlings that are correlated to a generation of high H_2_O_2_ which is comparable with previous findings (Hu et al., 2009; Hasanuzzaman et al., 2012a). The Cd has an affinity to proteins or –SH compounds and other side chains, Cd disturbs protein and enzymes synthesis which impair enzymatic activity (Sanitá di Toppi and Gabbrielli, 1999). CAT and GPX activities were restored and increased in H_2_O_2_ pretreated Cd-stressed seedlings which played vital roles in reducing the H_2_O_2_ level in Cd affected seedlings. Similar roles of H_2_O_2_ were observed in Cd affected rice seedlings (Hu et al., 2009), cucumber plants subjected to low light stress (Zhang et al., 2011) and in salt affected rice seedlings (Li et al., 2011).

Like other abiotic stresses, MG is overproduced within the plants under Cd stress (Hasanuzzaman et al., 2012a; Kaur et al., 2015; Nahar et al., 2015). Cd decreased both the activities of Gly I and Gly II that indicated the reduced MG detoxification via the glyoxalase system (Nahar et al., 2015). In this study, rapeseed seedlings exposed to Cd decreased Gly I and Gly II activities but pretreatment with H_2_O_2_ increased Gly I and Gly II activities and GSH content indicating the imperative roles of H_2_O_2_ in MG detoxification which are in the same line with the previous findings (Hasanuzzaman et al., 2012a; Nahar et al., 2016a,b).

## Conclusion

In this study, we provided evidence for a specific pattern of ROS generation (H_2_O_2_ and $O_{2}^{•–}$) and oxidative damage (MDA content) with the raise of Cd dose. Exogenous H_2_O_2_ treatment increased the amount of the most important ROS scavenging molecules AsA and GSH and increasing the antioxidant enzyme activities which enhanced ROS scavenging process. The Gly I and Gly II activities and content of GSH increased after H_2_O_2_ pretreatment indicating the roles of exogenous H_2_O_2_ in MG detoxification process. In contrast to the evidence of exogenous H_2_O_2_-induced ROS and MG detoxification in the present study, a number of unanswered questions still remain unclear. Why and how does H_2_O_2_ induce production of antioxidant molecules (AsA and GSH) and activities antioxidant enzymes? Previous reports support the notion that H_2_O_2_ induced signaling is involved with phytohormones and signaling molecules ABA, SA, JA (jasmonic acid), GA (gibberallic acid), ethylene, NO, Ca^2+^-mediated development of abiotic stress tolerances in plant (Mittler et al., 2004; Jubany-Mari et al., 2009; Chen et al., 2012; Guo et al., 2014; Reczek and Chandel, 2015; Saxena et al., 2016). In relation to the findings of present study, new questions arise: is there any signaling function of H_2_O_2_ in regulating the biosynthesis or degradation/metabolism of antioxidants components or, other hormones or signaling molecules affecting these processes? The possible mechanisms and signaling action of H_2_O_2_ in these aspects should be further elucidated.

## Author Contributions

MH, MF, and KN conceived and designed the experiments; MH and KN performed the experiments; HA and BR analyzed the data; MF contributed reagents/materials/analysis tools; MH, KN, SG, HA, and BR wrote the manuscript. BR edited the manuscript. All authors read and approved the final manuscript.

## Conflict of Interest Statement

The authors declare that the research was conducted in the absence of any commercial or financial relationships that could be construed as a potential conflict of interest.

## Abbreviations

AO: ascorbate oxidase
APX: ascorbate peroxidase
AsA: ascorbic acid (ascorbate)
BSA: bovine serum albumin
CAT: catalase
CDNB: 1- chloro-2, 4-dinitrobenzene
Chl: chlorophyll
DHA: dehydroascorbate
DHAR: dehydroascorbate reductase
DTNB: 5,5′-dithio-bis (2-nitrobenzoic acid)
EDTA: ethylenediaminetetraacetic acid
Gly I: glyoxalase I
Gly II: glyoxalase II
GPX: glutathione peroxidase
GR: glutathione reductase
GSH: reduced glutathione
GSSG: oxidized glutathione
GST: glutathione *S*-transferase
HSD: honest significant difference
LOX: lipoxygenase
MDA: malondialdehyde
MDHA: monodehydroascorbate
MDHAR: MDHA reductase
MG: methylglyoxal
NADPH: nicotinamide adenine dinucleotide phosphate
NTB: 2-nitro-5-thiobenzoic acid
PEG: polyethylene glycol
Pro: proline
ROS: reactive oxygen species
RWC: relative water content
SLG: *S*-D-lactoylglutathione
TBA: thiobarbituric acid
TCA: trichloroacetic acid
